# Apparent Young’s Modulus of Epoxy Adhesives

**DOI:** 10.3390/ma15228060

**Published:** 2022-11-15

**Authors:** Kamil Anasiewicz, Józef Kuczmaszewski

**Affiliations:** Department of Mechanical Engineering, Lublin University of Technology, 20-618 Lublin, Poland

**Keywords:** adhesive bonding, apparent Young’s modulus, adhesive joint zones

## Abstract

This article presents the results of a study of the properties of epoxy adhesives in an adhesive joint. The study analysed changes in Young’s modulus values as a function of the rigidity of the adhesive and the type of joined material. The values of Young’s modulus values were determined on the thickness of the adhesive joint using the nanoindentation method and in a tensile test of dumbbell shape sample for the adhesive material. The obtained results were analysed in terms of changes to the values of Young’s modulus of the adhesive as a function of the distance from the joined material–adhesive phase boundary and compared to the adhesive material. Zones were distinguished in the layer of the adhesive joint—adjacent to the wall and the core, with different values of Young’s modulus. Conclusions were drawn, indicating the relationship between the adhesive joint thickness and the increase in the value of Young’s modulus. Significant differences were found in the values of Young’s modulus of the adhesive joint compared to Young’s modulus of the adhesive in the form of plastic.

## 1. Introduction

In the preparation of this paper, a study intended to compare the properties of joints made using popular epoxy adhesives was performed. This study is a continuation of a series of publications by the authors dedicated to observations of the apparent Young’s modulus phenomenon in the changes to the properties of adhesive joints and the impact of these changes on the approach to numerical modelling of adhesive joints [1,2,3]. On the basis of the results of past research, differences were observed in the values of Young’s modulus in the adhesive joints compared to the properties of the adhesive as a material, without direct contact with the joint material during adhesive curing. A general conclusion can be formulated on the basis of these studies, namely that the adhesive joint metal–epoxy adhesive–metal can be interpreted within the area of the adhesive joint as a composite material, i.e., a material, the properties of which are not the sum or the average of those of its components. In order to support these conclusions, the authors decided to expand the scope of the research by studying a larger number of adhesives, joined materials, and thicknesses of adhesive joints.

The strength of the adhesive joints and their behaviour upon destruction is not only a complex topic, but they are also strongly correlated to the mechanical properties of the adhesive layer and the strain within the adhesive joint [4,5]. This is why many studies dedicated to the explanation of critical factors influencing the reliability and integrity of adhesive joints can be found in the literature. Studies available in the literature cover, inter alia, the impact of joint geometry, joint thickness, joint rigidity, taper, application rate, load, and temperature, as well as environmental conditions on the strength of the adhesive joint. The rigidity of the joined materials is a very important factor affecting the strength of the joint. Kinloch [4] provided a definition of the apparent Young’s modulus, defined as the ratio between the applied shearing stress and the strain at the thickness of the adhesive joint, which is greater than the actual Young’s modulus of the adhesive joint. The increased value of Young’s modulus results from the assumption that the adhesive material in the adhesive joint is strongly bound to the substrate of the stiff material of the joint. If we assume that the adhesive is ideally bound to the substrate and the substrate is ideally stiff, radial and circumferential strain in the joint are simultaneously equal to zero, and the value of the apparent Young’s modulus may be expressed using the formula:

$$E_{a}^{\prime }=\left[\frac{\left(1-v_{a}\right)}{\left(1+v_{a}\right)\left(1-2v_{a}\right)}\right]\cdot E_{a}$$

where: *E′_a_*—apparent Young’s modulus of the adhesive, *E_a_*—Young modulus of the adhesive, *v_a_*—Poisson’s ratio.

Construction adhesives achieving a Poisson’s ratio value of 0.35 according to the above formula could achieve a Young’s modulus value of the adhesive bond even up to 60% higher than the value of the Young’s modulus of the adhesive material [4]. However the provided formula does not present the relationship between the value of the apparent Young’s modulus and the thickness of the adhesive joint, the precise relationship between the changes in the adhesive joint and the distance from the phase boundary, as well as potential changes to the value of the Poisson’s ratio.

Researchers have often undertaken the topic of the influence of the adhesive joint thickness on single-overlap and double-overlap joints of metal sheets, using analytical calculations and the finite element method. The results of these studies allow the conclusion that the maximum strength of overlap joints joined using flexible adhesives increases with the decreasing thickness of the joint [6]. Other studies presented in the available literature rather narrowly address the topic of changes to adhesive properties in the adhesive joint during joint constitution. The scope of these studies often referred to a comparison between the strength of the adhesive joint and the strength of an adhesive joint in the form of a cast dumbbell shape sample [7]. It should be emphasised that such a comparison is difficult to make because of the complex adhesive and cohesive interactions within the joint [4,8,9]. In adhesive material, cohesion forces are responsible for the strength, whereas adhesive failure is very common in an adhesive joint. This means that cohesion forces are greater than adhesion forces determining the adhesion of the adhesive to the substrate. In order to intensify the influence of the cohesive strength of the joint, samples were prepared as a stack of butt joints, bonded alternately. In a sample prepared in this manner, there are several or dozen joints, deformations of which accumulate during the axial tension test. The preparation of such samples is problematic because of the need to ensure extremely precise preparation of many joined surfaces and of the problem with maintaining equal joint thickness throughout the entire stack. Appropriate geometric accuracy of the adhesive bond should also be preserved, i.e., stack linearity. The stack was destroyed in the chuck of a strength testing machine using an extensometer, and the results were compared to a tensile test of adhesive material in the form of a cast dumbbell shape sample. As a part of the results processing, the Young’s modulus of the adhesive material and of the adhesive joint were determined. These studies indicate small differences in the values of the Young’s modulus for the material and for the joint. It was also concluded that the joined material and the related interaction between joint surfaces might be an important factor influencing changes in the properties of the adhesive joint during curing. In the studies reported in [10], the authors investigated the effect of joint thickness on angular joints of composite connectors joined using a two-component construction adhesive. In their conclusions, the authors pointed out that the decrease in strength at failure with increasing thickness of adhesive weld, the small joint thickness facilitates the formation of a flat stress condition, while higher joint thicknesses facilitate flat deformation conditions. Subsequent studies compared the behaviour of adhesive in a joint and in an adhesive material in the dumbbell shape samples. On the basis of the results of this study, it was concluded that the observed differences in the apparent shear and tensile strength of adhesive joints between their in situ thin layer forms and adhesive material samples were caused by differences in the stress condition in sample configurations and not by differences related to material properties. It was also concluded that the mechanical properties of adhesives, determined on the basis of tests performed on adhesive samples in the material form, can be used in the design and analysis of joined structures [11]. In this case, however, the range of joint thicknesses studied is important, which included the thicknesses of 0.25 mm, 1.3 mm, and 2.5 mm. Further research involves studies on the physico-chemical and mechanical properties of aluminium substrates joined using commercially available epoxy adhesives. Adhesive joints were prepared in several thickness variants. The authors observed a slight deterioration of the mechanical properties of joints with increasing joint thickness. They also explained this property by a change to the stress condition during the testing of modified Arcana-type fixtures with a relatively thick layer of adhesive as a part of the results of numerical analysis [12]. The strength of adhesive joints generally increases with decreasing joint thickness [13]. Park et al. [14] tested thick aluminium overlap joints with four different adhesive layer thicknesses and predicted their strength based on the modified failure zone factor method. According to their experimental results, the failure load of overlap joints increases with joint thickness, increasing from 0.15 to 0.45 mm, and then gradually decreases when the joint thickness reaches 0.9 mm. Other studies have also compared the effect of joint thickness in a butt joint on the strength of the adhesive joint [15]. The results of these studies indicated a trend of joint strength degradation with increasing thickness of the adhesive joint. This can be attributed to the widely understood strengthening of the adhesive joint in a smaller volume of the adhesive. The increased strength of the adhesive joint with a lower joint thickness is certainly also influenced by the statistically smaller number of defects present in the smaller volume (thickness) of the adhesive joint. In the study performed in [16], the authors addressed the effect of joint thickness on tensile resistance to brittle fracture of adhesive joint samples with aluminium alloy in various thickness ranges. Subsequent studies [11,17] presented results indicating that in the case of a plastic epoxy adhesive, in an adhesive butt joint of the Iosipescu-type sample subjected to stretching, in which the stress is triaxial—not only is the apparent Young’s modulus of the adhesive joint increased, but also the butt joint strength. On the other hand, the present stress concentration reduces the failure stress of the butt joint bonded using a brittle epoxy adhesive.

In a summary of the presented studies, we can conclude that the phenomenon described as an apparent Young’s modulus of an adhesive joint strictly depends on the joint thickness, adhesive stiffness, joint configuration, and even on the method of use of the adhesive joint. All the presented research publications should certainly be analysed in terms of the phenomena occurring in the joint when the connection is being constituted, phenomena related to adhesion, and interactions between the surfaces of elements joined with adhesive in a liquid state. The results of the presented studies often indicated discrepancies in the strength of the adhesive joint in relation to the strength of the adhesive material; however, these differences were closely correlated with the thickness of the adhesive layer and with the connecting material. Taking into account the results of the studies presented above, the authors decided to investigate whether the material of the adhesive joint can be treated as a material with homogeneous properties, in relation to various epoxy adhesives, in terms of their stiffness, joint thickness, and two selected bonded materials. The aim of the presented research is to precisely define the material properties of the adhesive material in the adhesive joint.

## 2. Materials and Methods

Three epoxy resins: Epidian 5, Epidian 57, and Epidian 6, were selected for the study. Epoxy adhesives and hardeners were manufactured by Sarzyna Chemical Ltd. (Nowa Sarzyna, Poland). Epidian epoxy resins are popular engineering adhesives used in the industry, including aviation and automotive. Two hardeners, designated PAC and Z1, were used in the preparation of the adhesive mixture. AW 2024-T3 aluminium alloy sheets and 1.4301 stainless steel sheets were the joined materials. These materials were selected for the study due to their high popularity in the aviation and automotive industries and the fact that the phenomenon of apparent Young’s modulus should be more emphasized when joining rigid materials. Due to one of the objectives of the research, i.e., the observation of differences in the value of Young’s modulus of the adhesive joint depending on its stiffness, two hardeners, PAC and Z1 were selected for investigation. Mixing the PAC hardener with the epoxy resin results in an elastic epoxy adhesive, whereas mixing the Z1 with the epoxy resin results in obtaining stiffer epoxy adhesive. The main components of the epoxy resins and hardeners with mass ratios of the used adhesive mixtures are shown in Table 1.

**Figure 1:**
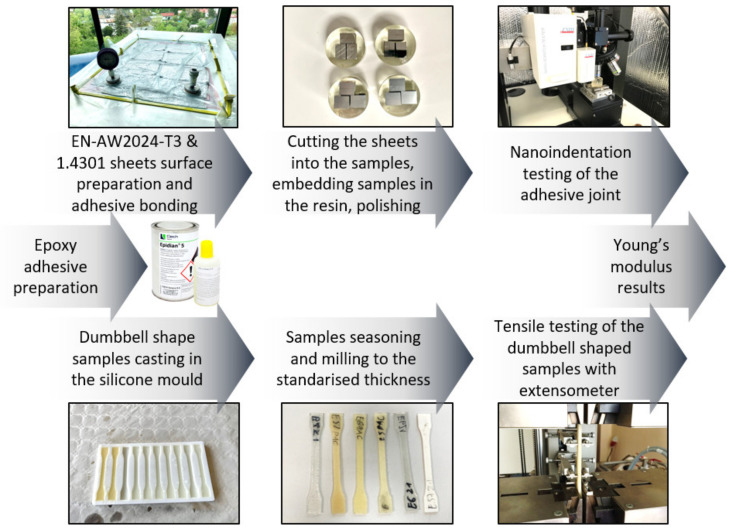
Schematic diagram of the testing procedure.

Figure 1 shows a schematic diagram of the testing process. Twelve samples—two for each adhesive mixture and one for each of the joined materials, respectively—were prepared as two bonded sheets with the initial dimensions of 40 × 60 mm. The thickness of the aluminium alloy sheets was 5 mm, and the thickness of the stainless-steel sheets was 4 mm. The sheets were cut using a hydroabrasive jet. The surface of the sheets was cleaned with a cleaning agent and a rinsing agent. The surface of the stainless-steel sheet was sanded manually using 320-grit sandpaper, with circular motions intended to produce a random direction of surface roughness. In the case of surface preparation for sheets made of aluminium alloy, P320 non-woven fabric was used, and the surface was ground in such a manner to ensure a random distribution of unevenness on the sheet surface. Once the surface of the sheets was prepared by grinding, the surface was cleaned using a cleaning agent and twice using the rinsing agent Loctite 7061. The excess was wiped using the cleaning agent and the sample was left to dry. The prepared adhesive compositions were mixed for 3 min, using a mechanical stirrer at 400 rpm, changing the direction of rotation of the stirrer 6 times. After mixing, the mixture was degassed for 5 min using a vacuum pump in order to remove air bubbles from the adhesive volume. The adhesive composition prepared in this manner was applied onto the cleaned surface of the sample, using a spatula, distributing the adhesive uniformly on both surfaces to be joined. The sheets were joined, secured against moving, and placed in a vacuum bag using a vacuum of 0.1 MPa. Different joint thicknesses were achieved due to the varying density of the adhesive mixture. An attempt was made to obtain the lowest possible joint thicknesses in order to observe the strongest possible ‘strengthening’ in the area of the adhesive joint.

**Figure 2:**
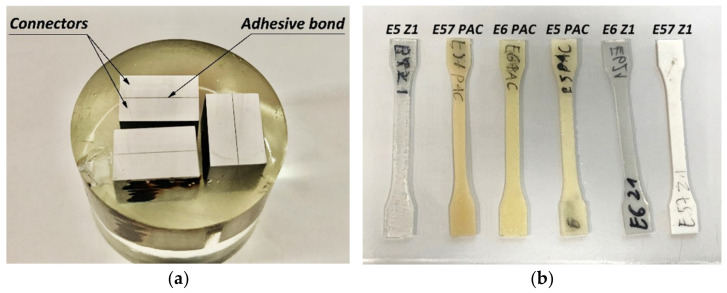
Samples of the adhesive bonds prepared for nanoindentation testing (**a**), dumbbell-shaped samples for tensile testing (**b**).

Samples subjected to the pressure of 0.1 MPa were cured for a period of 24 h at 18–20 °C and at 38–40% relative humidity, and subsequently, after removal from the vacuum bag, they were seasoned for 168 h. At the end of this period, 10 × 15 mm samples for nanoindentation tests were cut from the middle of the sheet. The samples were placed in moulds and cast over with resin, with the testing surface exposed. The samples were prepared by initial grinding on grinding discs while intensive cooling was applied simultaneously. Surface preparation was continued using sandpaper, starting with a very coarse grain (180, 240), while very precise preparation used sandpaper with a fine grain (1000, 1200). Conditions for keeping thermal influence on the adhesive joint to a minimum were maintained during the grinding. After the grinding, the samples were polished mechanically on horizontally positioned, felt-lined rotary discs coated with an Al_2_O_3_ aqueous suspension. Polishing was carried out until a mirror-like, scratch-free surface was achieved. The finished sample was washed in water and ethanol and then dried in a stream of compressed air. Samples prepared for nanoindentation and exemplary dumbbell-shaped samples are shown in Figure 2.

**Figure 3:**
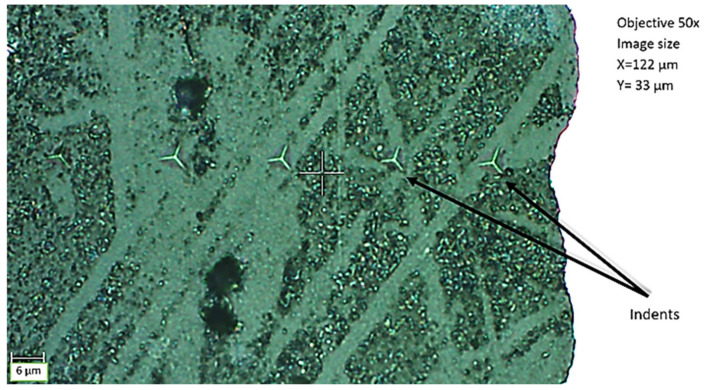
The example of an adhesive bond with visible nanoindentations.

A test intended to determine the differences in values of the Young’s modulus on adhesive thickness as a function of the distance from the phase boundary was performed using a CSM Instruments ultrananoindenter, manufactured by Anton Paar GmbH, Graz, Austria. The device enables microhardness measurements and calculations of hardness and of the Young’s modulus using the Olivier–Pharr method with high accuracy [18]. The accuracy of the indenter positioning during the measurement is 0.25 µm, whereas the measurement of the applied force can be carried out with a resolution of 0.005 µN. Nanohardness is defined as a measure of the resistance of a material when an indenter with a defined geometry and well-defined properties of its material is inserted perpendicular to its surface. The test was carried out on the thickness of the adhesive joint, starting from the metal–adhesive phase boundary to the centre of the joint, using a reference head that enables constant indenter insertion depths to be maintained in relation to the surface of the material. Indentations were made at 5 points, starting at a distance of 3 µm, 6 µm, 9 µm, in the middle of the joint thickness and a point between the middle of the joint and the 9 µm point. The use of this measurement method was based on the previous research experience, showing that potentially the largest changes in the values of Young’s modulus can be expected within a distance of 9–10 µm from the phase boundary; therefore, the authors decided to make more indentations in the boundary zone. Due to the fact that the main objective of the research was not to obtain an exact distribution of the Young’s modulus on the thickness of the adhesive joint, but to determine the differences in the value of the Young’s modulus of the adhesive in direct contact with the joined material and the adhesive located in the central part of the joint, it was decided to vary the distance of the measurements. Indentations were made down to half of the thickness of each joint. To ensure result averaging, 10 repetitions were made for each of the measurement positions at various points of the adhesive joint. Nanoindentation was performed using a diamond indenter with Berkovich geometry to a fixed depth of 800 nm. The first of the measurements was performed as close to the metal–adhesive boundary as possible, at a distance of 0.003 mm. Because of the need to maintain an adequate distance between individual indentations, in order to eliminate the impact of subsequent indentations on one another, the indentations were offset at an angle instead of placing them perpendicular to the joined elements. Offsetting the indentations by an angle instead of placing them on one line is a standard procedure. It prevents individual indentations from interacting with each other, which could otherwise distort the measurement results. Figure 3 shows an example of an adhesive bond with visible nanoindentations.

The excess of the prepared adhesive composition was cast in a dumbbell-shaped silicon mould simultaneously with the preparation of the butt-joined samples. The samples were left to cure for a period of 7 days. At the end of this time, the samples were milled to bring them to a uniform thickness of 4 mm. The samples prepared in this manner were subjected to an axial tensile test intended to determine the Young’s modulus. The test was performed in accordance with the DIN EN ISO 527-1 standard [19] using an extensometer ensuring precise measurement of sample elongation over a distance of 50 mm. The stress–strain characteristics and the failure strength of the adhesive material were also determined in this tensile test. The values of the Young’s modulus of adhesive materials were determined on the basis of the obtained elongation curves. The nanoindentation method, as the Young’s modulus determination method, was validated by determining the Young modulus using this method on adhesive material and comparing it to the value determined on the basis of a tensile test of a dumbbell shape sample using an extensometer. The results obtained for both methods are similar.

## 3. Results

**Figure 4:**
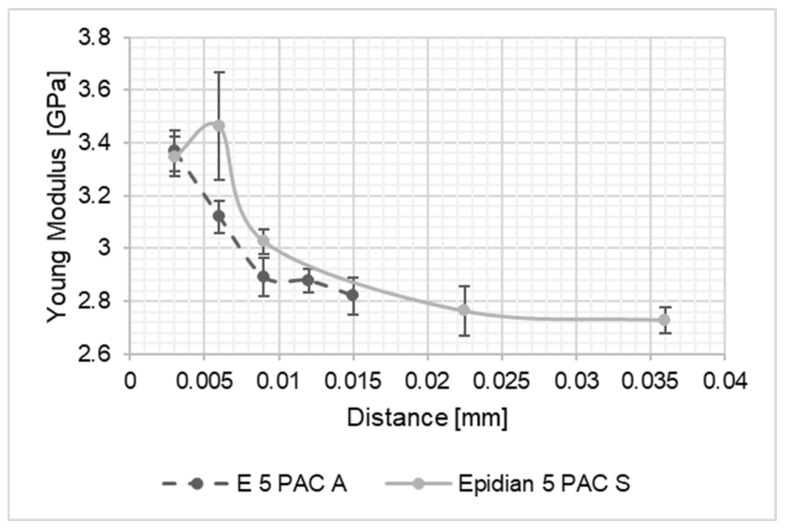
Comparison of Young’s modulus values for the Epidian 5/PAC joint bonding: A—EN-AW 2024-T3 aluminium alloy sheets; S—1.4301 stainless steel sheets.

**Figure 5:**
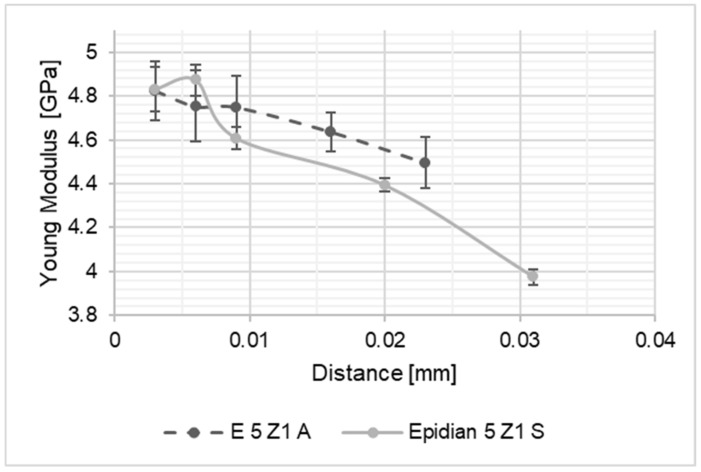
Comparison of Young’s modulus values for the Epidian 5/Z1 joint bonding: A—aluminium alloy sheets; S—stainless steel sheets, as above.

**Figure 6:**
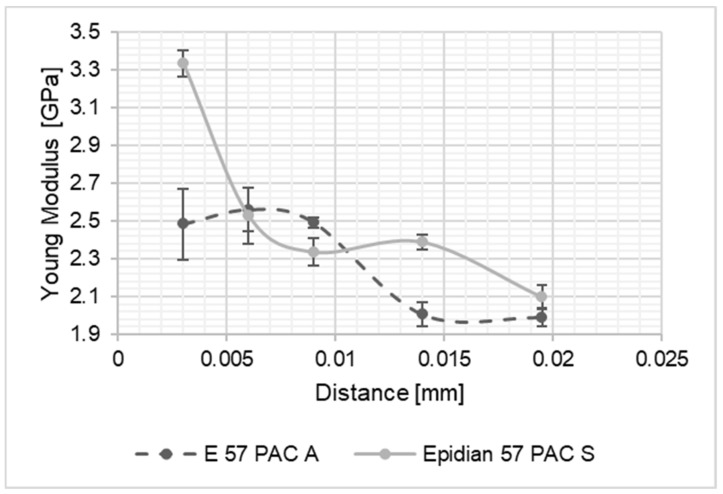
Comparison of Young’s modulus values for the Epidian 57/PAC joint bonding: A—aluminium alloy sheets; S—stainless steel sheets, as above.

**Figure 7:**
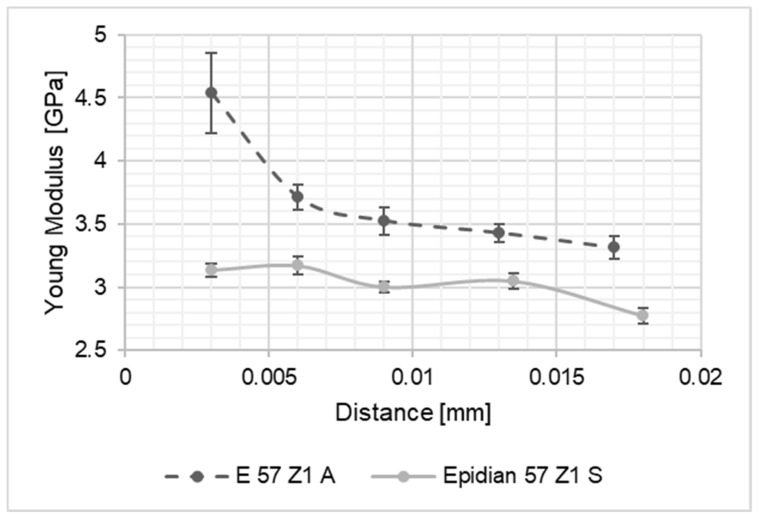
Comparison of Young’s modulus values for the Epidian 57/Z1 joint bonding: A—aluminium alloy sheets; S—stainless steel sheets, as above.

**Figure 8:**
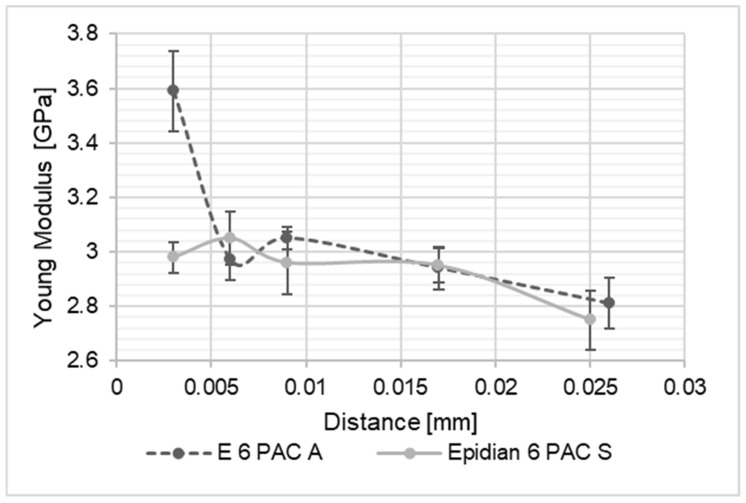
Comparison of Young’s modulus values for the Epidian 6/PAC joint bonding: A—aluminium alloy sheets; S—stainless steel sheets, as above.

**Figure 9:**
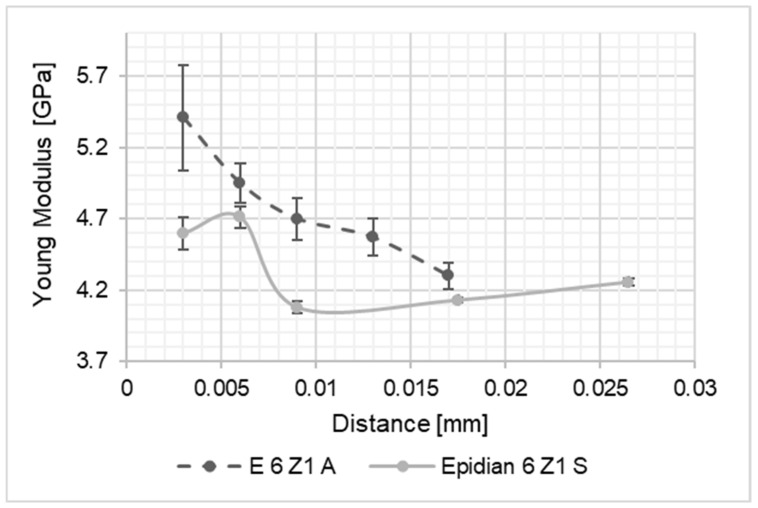
Comparison of Young’s modulus values for the Epidian 6/Z1 joint bonding: A—aluminium alloy sheets; S—stainless steel sheets, as above.

As a result of the performed studies, results intended to identify the differences in material properties of the adhesive joint along its thickness were processed. The thus obtained values of the Young’s modulus of the joints were compared to one another and with the values of Young modulus of the adhesive material, determined on the basis of tensile tests of the adhesive material in the form of the dumbbell shape samples. The graph presents a summary of the results for the Young’s modulus values along the joint thickness. In the diagrams shown in Figure 4, Figure 5, Figure 6, Figure 7, Figure 8 and Figure 9, the distance from the metal–joint phase boundary is indicated on the horizontal axis, starting from 0 to the measurement point in the middle of the joint.

Table 2 presents Young’s modulus values for epoxy adhesives in the form of a cast material.

**Figure 10:**
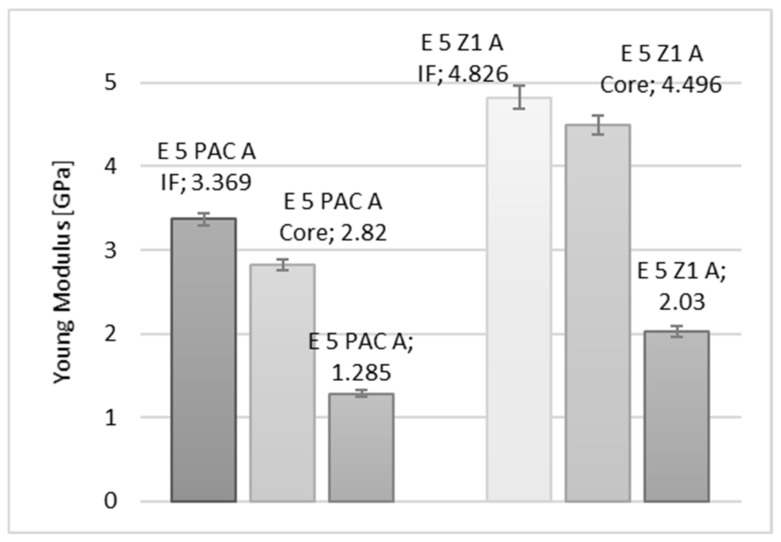
Comparison of the values of the Young’s modulus of an Epidian 5/PAC, Epidian 5/Z1 adhesive joint, respectively, for a joint in the wall-adjacent zone—IF, the core—Core, and for the adhesive material.

**Figure 11:**
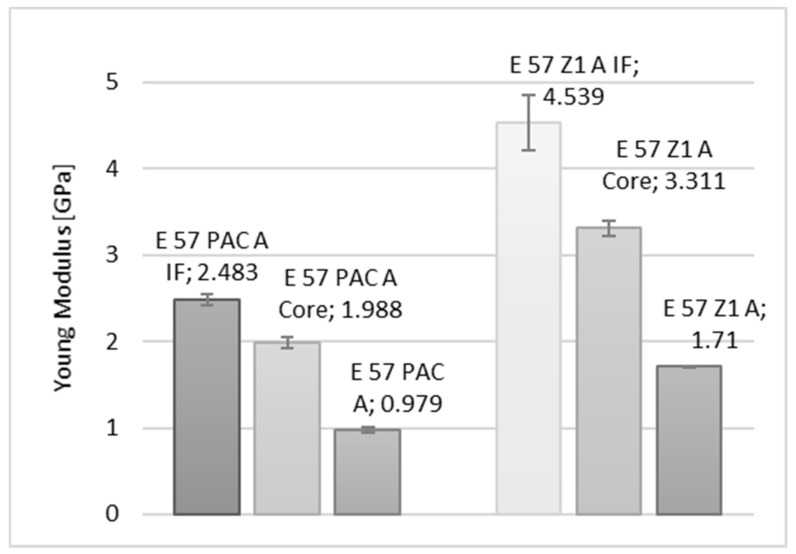
Comparison of the values of the Young’s modulus of an Epidian 57/PAC, Epidian 57/Z1 adhesive joint, respectively, for a joint in the wall-adjacent zone—IF, the core—Core, and for the adhesive material.

**Figure 12:**
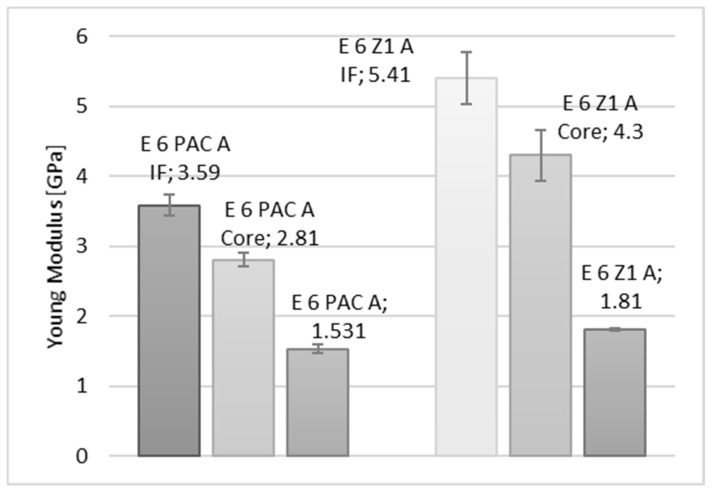
Comparison of the values of the Young’s modulus of an Epidian 6/PAC, Epidian 6/Z1 adhesive joint, respectively, for a joint in the wall-adjacent zone—IF, the core—Core, and for the adhesive material.

In the diagrams shown in Figure 10, Figure 11 and Figure 12, a comparison of the values of the Young’s modulus for three reference measurement points is provided: Wall-adjacent zone (IF), joint core (Core), and cast sample (-). The comparison is shown for samples with connected elements made of an aluminium alloy.

## 4. Discussion

By analysing the results presented above and taking into account the authors’ prior research, intended to determine the changes in the Young’s modulus of adhesive joints, we may notice that in the case of thinner joints, the Young’s modulus is higher for the joint material compared to the adhesive material.

This study analysed joints with thicknesses in the 0.035–0.07 mm range. Such joint thicknesses may be classified as very thin joints. On the basis of the study presented here and of the prior studies by the authors, we can conclude that the Young’s modulus of an epoxy adhesive joint with a thickness below 0.1 mm assumes values higher than for the adhesive material as such. A Young’s modulus value higher by up to 130% compared to the value of the Young’s modulus of the adhesive material cast as standardised dumbbell shape samples should be assumed. In the case of samples above the threshold of 0.1 mm in the joint, we can observe clear differences in the values of the Young’s modulus, the value of which changes along the joint thickness and in the core area of the joint; it may reach values similar to the values of the Young’s modulus corresponding to the adhesive material [1,3]. In thicker joints, it is possible to observe a clear coincidence of the Young’s modulus value of the joint core and of the adhesive material in the form of a cast dumbbell shape sample. A significant difference between those values is observed in thin joints. The value of the Young’s modulus in the wall-adjacent zone is ca. 15–25% higher than the value of the Young’s modulus in the zone identified as the joint core—the area located the farthest from the edge of the joint (of the joined material). The authors concluded that the differences in the values of Young’s modulus between the measurement points, reaching more than 15%, can be defined as zones with different properties and considered individually. In the case of this type of joint, the joint should be treated as having three layers to improve the simulation results. This allows a more accurate representation of the actual values, but it requires higher computational power and longer times for the simulation. Taking into account the standard deviations of the measurements, in each of the measured points for all adhesive joints, it should be indicated that the standard deviation is in the range of 0.9–7.7%. It is a much lower value when compared with the difference between the values of the Young’s modulus of the adhesive joint in the boundary zone and the adhesive joint core–centre of the joint.

In the case of stainless-steel joints, the results indicate a characteristic increase in the Young’s modulus at the second measurement point—6 µm from the phase boundary. The value of the Young’s modulus measured closest to the phase boundary, at a distance of 3 µm, is increased compared to the value of the Young’s modulus of the joint core: it is lower, however, than the value corresponding to the distance of 6 µm. The variation in values of the Young’s modulus in this area may indicate strong anisotropy between the wall-adjacent area and the joint core. We may conclude that a significantly higher cross-linking rate is present in the structure of the epoxy adhesive in the wall–adjacent area compared to the joint core. A general conclusion may be formed that the conditions of adhesive curing in the adhesive joint are different in the layer in contact with the bonded material—the wall-adjacent layer, compared to the adhesive core—the layer located the farthest away from the surface of the bonded material.

It should now be determined whether these differences have a significant influence on the approach to numerical modelling, which currently assumes the joint model as homogeneous—with the same value of the Young’s modulus within the entire volume of the adhesive joint.

Changes in the stress–strain characteristics of the adhesive joint, in relation to flexible interactions, do not necessarily mean an increase or a decrease in the strength of the adhesive joint for obvious reasons. Moreover, if the joint is considered as a material with heterogeneous properties present in the layers, it can be assumed in such an approach that layers will also be present in the joint (e.g., in the joint core), with properties similar to the adhesive material in the form of a cast material. The change in values of the Young’s modulus of the adhesive is mainly caused by the direct contact between the joint and the bonded material, with the value of the Young’s modulus 35 to 100 times higher than in the joint material. The observed changes in properties of the adhesive joint clearly influence the rigidity of the connection and the nature of its use in terms of the flexible stress of the adhesive joint; however, it has no direct impact on the adhesive strength of the adhesive joint. The observed changes are related to the formation of the adhesive joint, and it may be assumed that this is a phenomenon that is a consequence of adhesive joint formation. Accurate observation of this phenomenon and identification of the areas where it is present is important from the point of view of forecasts related to the use of adhesive joints using numerical modelling methods.

## 5. Conclusions

The following conclusions were formulated as a result of the study:

- The value of the Young’s modulus of the adhesive joint material depends on the joint thickness and on the distance from the phase boundary.
- The value of the Young’s modulus of the adhesive joint measured using the nanoindentation method is different from the value of the Young’s modulus of the adhesive material. The Young’s modulus of the adhesive joint reaches values 85% to 135% higher than the values of the Young’s modulus of the adhesive material, with values in the wall-adjacent zone being as much as 240% higher. These values correspond to the values of the Young’s modulus obtained in past studies of the authors for a similar type of epoxy adhesives [2].
- Young modulus is standardised at a distance of 0–10 µm. In this range, the extent of interaction between the surface of the joined metal and the adhesive joint rapidly decreased.
- Joints with thickness above 0.05 mm should be treated as having three zones distinguished within them.
- Due to the small thickness of the joint, joints with thickness below 0.05 mm may be treated as uniform, with the difference, however, that its material properties, and the Young’s modulus, in particular, differ from values measured for the adhesive material under conditions without contact with the target material, e.g., the metal sheet.
- In bonded samples using stainless steel, a characteristic increase in the Young’s modulus can be observed at the second measurement point—6 µm, for two out of three adhesives. This is not a significant increase, but clarification of its nature requires more extensive studies of the microstructure.
- We can observe differences, which depend on the type of adhesive used when comparing the values of the Young’s modulus for various joint materials. In the case of the Epidian 5/PAC and Z1 adhesive, the values of the Young’s modulus values reach a similar level, regardless of the type of the bonded material. In contrast, in the case of the Epidian 6/PAC and Z1 adhesive, the values of the Young’s modulus for both adhesives joining connected elements made of aluminium alloys are ca. 20% higher in the wall-adjacent zone compared to joints bonding elements made of stainless steel. In the core area of the joint, the values reach similar levels, regardless of the type of bonded material.
- A comparison of joints made using a flexible adhesive (with added PAC) and a rigid adhesive (with added Z1) indicated a clearer division of the joint into zones, if a flexible adhesive was used.

## Figures and Tables

**Table 1:** Table of main components of epoxy resins and hardeners with weight ratios of the used adhesive mixtures.

Epoxy Resin	Hardener [g/100 g]	
	PAC—Polyaminoamide—Based, Main Components: Fatty Acids, C18-Unsaturated, Dimers	Z1—Aliphatic Amine—Based, Main Components: Triethylenetetramine
Epidian 5—base unmodified liquid resin, BPA—Based. Bisphenol A and epichlorohydrin.	80	12
Epidian 57—BPA—based contains bisphenol A and epichlorohydrin, polyester diluent.	65	10
Epidian 6—base unmodified liquid resin, BPA -based, contains: hydro—lyzable, chlorine cresyl glycidyl ether and benzyl alcohol.	80	12

**Table 2:** Average Young’s modulus values determined in a tensile test of the adhesive material.

Adhesive	Average Young’s Modulus [GPa]	Standard Deviation [GPa]
Epidian 5/PAC	1.285	0.09
Epidian 5/Z1	2.03	0.13
Epidian 57/PAC	0.979	0.057
Epidian 57/Z1	1.7	0.024
Epidian 6/PAC	1.531	0.12
Epidian 6/Z1	1.81	0.16

The above values of the Young’s modulus may be interpreted as corresponding to the Young’s modulus of the core of the adhesive joint for joints reaching 0.1 thickness and thicker.

## Data Availability

The data presented in this study are available on request from the corresponding author.

## References

[1] Anasiewicz K., Kuczmaszewski J. (2019). Adhesive Joint Stiffness in the Aspect of FEM Modelling. Materials.

[2] Anasiewicz K., Kuczmaszewski J. (2016). Apparent Young’s modulus of epoxy adhesives in metal bonding. Przegląd Spaw.

[3] Anasiewicz K., Kuczmaszewski J. (2021). Apparent Young’s Modulus of the Adhesive in Numerical Modeling of Adhesive Joints. Materials.

[4] Kinloch A.J. (1987). Adhesion and Adhesives Science and Technology.

[5] Wang C.-H., Rose L. (1997). Determination of triaxial stresses in bonded joints. Int. J. Adhes. Adhes..

[6] Zhu Y., Kedward K. (2005). Methods of Analysis and Failure Predictions for Adhesively Bonded Joints of Uniform and Variable Bondline Thickness.

[7] Godzimirski J., Tkaczuk S. (2004). Określanie Właściwości Mechanicznych Spoin Klejowych. Technol. I Autom. Montażu.

[8] Adams R.D. (1997). Structural Adhesive Joints in Engineering.

[9] Kuczmaszewski J. (1995). Podstawy Konstrukcyjne I Technologiczne Oceny Wytrzymałości Adhezyjnych 269 Połączeń Metali.

[10] Taib A.A., Boukhili R., Achiou S., Gordon S., Boukehili H. (2006). Bonded joints with composite adherends. Part I. Effect of specimen configuration, adhesive thickness, spew fillet and adherend stiffness on fracture. Int. J. Adhes. Adhes..

[11] Air Traffic Organization Operations Planning Office of Aviation Research and Development (2008). 279 Investigating the Thin-Film Versus Bulk Material Properties of Structural Adhesives.

[12] Davies P., Sohier L., Cognard J.-Y., Bourmaud A., Choqueuse D., Rinnert E., Créac’Hcadec R. (2009). Influence of adhesive bond line thickness on joint strength. Int. J. Adhes. Adhes..

[13] Afendi M., Teramoto T. (2009). Effect of Bond Thickness on Fracture Behavior of Interfacial Crack in Adhesive Joint of Dissimilar Materials. J. Adhes. Soc. Jpn..

[14] Park J.-H., Choi J.-H., Kweon J.-H. (2010). Evaluating the strengths of thick aluminium -to- aluminum joints with different adhesive lengths and thicknesses. Compos. Struct..

[15] Anasiewicz K., Kuczmaszewski J. (2017). Wpływ grubości warstwy kleju na sztywność spoiny w połączeniu doczołowym metal-metal. Technol. I Autom. Montażu.

[16] Lee D.-B., Ikeda T., Miyazaki N., Choi N.-S. (2002). Damage zone around crack tip and fracture toughness of rubber-modified epoxy resin under mixed-mode conditions. Eng. Fract. Mech..

[17] Adams R.D., Coppendale J. (1979). The Stress-Strain Behaviour of Axially-Loaded Butt Joints. J. Adhes..

[18] Oliver W.C., Pharr G.M. (2004). Measurement of hardness and elastic modulus by instrumented indentation: Advances in understanding and refinements to methodology. J. Mater. Res..

[19] (2012). Plastics—Determination of Tensile Properties—Part 1: General Principles.

